# A neonatal murine model for evaluation of enterovirus E HY12 virus infection and pathogenicity

**DOI:** 10.1371/journal.pone.0193155

**Published:** 2018-02-15

**Authors:** Xiaochun Gai, Qun Zhang, Haibing Lu, Zhanqing Yang, Lisai Zhu, Xin Li, Xinping Wang

**Affiliations:** 1 College of Veterinary Medicine, Jilin University, Changchun, China; 2 Key laboratory for Zoonosis, Ministry of Education, Institute of Zoonosis of the Jilin University, Changchun, China; The University of Chicago, UNITED STATES

## Abstract

**Backgrounds:**

HY12 viruses are enteroviruses recently isolated from cattle characterized by severe respiratory and digestive disease with high morbidity and mortality in China. While the viruses exhibit unique biological and molecular characters distinct from known enterovirus E, the pathogenicity and viral pathogenesis remains largely unknown.

**Methods:**

Neonatal mice of Balb/C, ICR, and Kunming strain are infected with HY12 to determine the susceptible mouse strain. The minimal infection dose, the virus infection routes, the pathogenicity and tissue tropism for HY12 were determined by infecting susceptible mice with HY12 viruses, and confirmed by different approaches including virus isolation and recovery, virus detection, histopathology, and immunohistochemistry.

**Results:**

A murine model for HY12 infection was successfully established and employed to investigate the pathogenicity of HY12 viruses. ICR mouse strain is the most susceptible strain for HY12 infection with a minimal infective dose as 2×10^6^TCID_50_/mouse. HY12 viruses have the capability of infecting ICR suckling mice via all infection routes including intranasal administration, oral administration, intraperitoneal injection, subcutaneous injection, and intramuscular injection, which are confirmed by the isolation and recovery of viruses from HY12-infected mice; detection of viruses by RT-PCR; observations of pathological lesions and inflammatory cell infiltrations in the intestine, lung, liver, and brain; uncovering of HY12 virus antigens in majority of tissues, especially in intestine, lung, and infected brain of mice by immunohistochemistry assay.

**Conclusions:**

A neonatal murine model for HY12 infection is successfully established for determining the susceptible mouse strain, the minimal infective dose, the infection route, the viral pathogenicity and the tropism of HY12, thus providing an invaluable model system for elucidating the pathogenesis of HY12 viruses and the elicited immunity.

## Introduction

The genus *Enterovirus* within the family of *Picornaviridae* contains 9 species enteroviruses (A, B, C, D, E, F, G, H and J) and 3 species rhinoviruses (A, B, C) according to the latest virus taxonomy [1]. Those viruses are etiologically related to respiratory and digestive diseases in human and animals [2–7]. Out of 9 enterovirus species, enterovirus E (EV-E), enterovirus F (EV-F) and enterovirus G (EV-G) are responsible for infections affecting livestock industry [2–4,8–11]. Bovine enteroviruses are composed of two enterovirus species, EV-E and EV-F, which are the causative agents of bovine enterovirus infection in cattle showing clinical signs from respiratory diseases to enteric, reproductive disease and infertility. Enterovirus G (EV-G), named porcine enterovirus B previously, is the causative pathogen of enterovirus infections in swine [5,12]. Recently, enterovirus infections in ovine/caprine were reported, and the viruses were showed to be different from EV-E, EV-F and EV-G [3,5,12–14]. EV-E and EV-F were frequently isolated from the cattle with symptoms of digestive and respiratory diseases indicate those viruses exhibit the pathogenicity to their hosts [4,8,11–13,15,16]. While the increasing number of enteroviruses isolated from animals developing respiratory and digestive diseases, the viral pathogenicity for enteroviruses remains largely unknown. Previously, we reported the isolation of an EV-E3 isolate HY12 associated with an outbreak characterized by respiratory and digestive diseases with morbidity and mortality up to 50%, and found that HY12 viruses possesses extremely high TCID_50_ titer with unique mutations in V1 and VP4 structural protein [4].

Animal model system remains the most effective means to investigate the pathogenicity and pathogenesis of microorganisms, and murine model system has been widely used in the studies of human enterovirus infection [17–21]. Although the best way to determine the pathogenicity of microorganism is to infect the animals where the pathogens are isolated, this system is not feasible for studying animal enterovirus infections, especially for the enteroviruses isolated from large animal mainly due to the paucity of animals with required backgrounds and the experimental expenditures. Therefore, establishment of a mouse model system will facilitate the studies of viral pathogenesis and immunity triggered by animal enteroviruses as such bovine enterovirus infection. In this study, we employ HY12 viruses to establish a murine model for bovine enterovirus infection, determine the susceptible mouse strain, the minimal infection dose and the infection routes, and define the tissue tropism and pathogenicity, thus providing an invaluable model system for determining the pathogenicity and exploring the mechanism underlying HY12 virus infection.

## Materials and methods

### Ethics statement

Mice and the procedures used for this study were following a standard protocol reviewed and approved by the Institutional Animal Care and Use Committee (IACUC) of Jilin University (approval no JLU-20150226), following the strict compliance with requirements of the Animal Ethics Procedures and Guidelines of the People′s Republic of China. Pregnant BALB/c, ICR and Kunming were obtained from the Institute of Changchun Biological Products. The mice were maintained in the Laboratory Animal Facility of Jilin Province. All mice had free access to food and water and were kept in a temperature-controlled room (22 ±0.5°C) on reverse 12/12 h light/dark cycle. The new-born pups aged at three days were randomly assigned to different treatment groups with each cage litter containing a dam and 5–10 pups.

### HY12 viruses and infection of neonatal mice

HY12 virus is an enterovirus E strain isolated from cattle characterized by severe diarrhea and respiratory disease with high morbidity and mortality [4]. The HY12 virus stocks of the 5^th^ passage with a known titer (10^12^ TCID_50_/ml) were kept at −80 °C and thawed at 4 °C before use, and pre-warmed to room temperature immediately prior to animal inoculation. Viruses used for inoculations were obtained by diluting the stock viruses with Dulbecco’s modified Eagle medium (DMEM, Thermo Fisher Scientific) in a total volume of 50 μl containing the corresponding TCID_50_ required by experiments.

Virus inoculations were performed inside a biosafety cabinet (Class II). For intramuscular injection and subcutaneous injection, 50 μl of viruses was injected into each mouse at two sites using a 31G ultra-fine hub-less insulin syringe (Beckton, Dickinson and Company, Franklin Lakes, New Jersey, USA). For intraperitoneal injection, 50 μl of viruses was administered. For intranasal and oral administrations, 50μl of viruses were delivered through a STD Mouse Jugular Vein Cath system (Access^™^ technologies, Skokie, IL, USA). Mice were acclimatized for 10 min prior to handling, inoculation, or observation.

### Necropsy, gross pathological observation, and tissue collection

Suckling mice were euthanized using cervical dislocation after CO2 inhalation. The euthanized mice were necropsied inside a Class II biosafety cabinet following the standard protocols. Tissue including liver, lung, spleen, lymph nodes, brain, and kidney of each mouse were collected and processed for virus recovery and virus detection by RT-PCR. Tissue samples used for histopathological examination and immunohistochemistry assay were collected separately and fixed in 10% neutral buffered formalin for 48 h at room temperature before they were kept in 75% ethanol.

### Tissue processing for pathohistological analyses

Tissue samples for histopathological analyses and immunohistochemistry assays were processed following standard procedure as previously reported[22]. Briefly, formalin-fixed tissues were dehydrated in a series of increasing concentrations of ethanol (70%, 80%, 95% and 100%) before they were incubated in Xylene (Thermo Fisher Scientific) two times with each time incubating for 1 h at room temperature, and then infiltrating with melted paraffin wax in an oven at 65 °C. Paraffin-embedded tissue blocks were sectioned at 5 μm using a microtome. The sections were loaded to polylysine-coated glass slides, dried overnight at 42 °C, and stored at room temperature for further use.

### Staining of tissue sections and immunohistochemistry assay

Hematoxylin and Eosin (H&E) staining was performed as previously described [22]. Briefly, tissue sections were dewaxed by incubation in xylene, and rehydrated in decreasing alcohol concentrations (100%, 95%, 70%, and 50%). Immunohistochemistry detection were performed following the procedures described. Briefly, after dewaxing and rehydration, slides were boiled for antigen retrieval in citrate buffer (pH 6.0) for 15 min, cooled to room temperature gradually, and then quenched the endogenous peroxidases by incubation of the slides in 3% H_2_O_2_ for 15 min at room temperature. After blocking with 1% bovine serum album for 30 min at room temperature, the slides were incubated with the polyclonal antibodies generated against HY12 encoded-VP2 recombinant proteins for 1 h at room temperature following by three washings in Tris-buffered saline (pH 7.4), 0.05% Tween-20 (TBS-T). The slides were then reacted with goat anti-rabbit Ig-HRP (Dako Cytomation, Glostrup, Denmark) for 30 min at RT. After washing, slides were incubated in diaminobenzidine (DAB) substrate, and counterstained with Hematoxylin. The lesions and immunostaining signals were graded as I, II, III mainly based on the severity of lesions and extents of the signals, and visualized and captured using a CCD camera mounted on a Nikon epifluorescence microscope (Nikon Instruments Co., Ltd, Shanghai).

### Cell culture and virus isolation

Vero cells were grown in Dulbecco’s modified Eagle medium (DEME) containing 10% fetal calf serum (FBS) (HyClone, Logan, UT) and maintained in DMEM containing 2% FBS. Virus isolation was performed as previously described [4]. Briefly, tissue samples from infected mice were homogenized in a dilution of 1:10 (W/V) with 10 mM phosphate buffered saline (PBS), centrifuged at 10,000 × g at 4°C for 10 min, then passed through 0.45 nm filter before inoculating to cells. Cell cultures were examined for the presence or absence of cytopathic effect (CPE) before they were frozen and thawed.

### Indirect immunofluorescence assay (IFA)

Vero cells were seeded into 24-well plates and infected with the recovered HY12 viruses from infected mice. The uninfected Vero cells were used as the negative controls. 24–48 h postinfection, cells were fixed with methanol/acetone (1:1) for 30 min at -20°C, blocked using 1% bovine serum albumin, and incubated with polyclonal antibody against HY12 VP2 recombinant protein for 1h at 37°C before the addition of Rhodamine-conjugated goat anti-rabbit IgG (1:500 dilution). After incubation for 45 min at 37°C, the cells were washed 3 times with PBS, sealed with glycerol, and examined using the fluorescence microscope.

### RNA isolation and RT-PCR

Total RNAs were extracted from either the tissue samples or infected cell cultures using TRNzol kit (Tiangen, Beijing) following manufacturer’s instructions. The resultant RNAs were kept at –80°C for further analysis. Reverse transcriptase reactions were performed using SuperScript^™^ II Reverse Transcriptase (Invitrogen, Carlsbad, CA) following the manufacture’s instruction. The cDNA synthesis was performed at 42 °C for 60 min. PCR amplification was done using Taq DNA polymerase (Takara, Dalian, Liaoning) according to manufacturer’s instruction. The primers used to detect enterovirus E were designed based on the nucleotide sequence of HY12 strain. The primer sequence was listed as fellows. HY12-UP: 5’- TAAGAAGTGCATGCCCCTAATC-3’; HY12-DN: 5’- AGAGACGCCGAAATCAAAG-3’.

### Quantitation of virus loads in mice infected with HY12 virus

Total RNA was extracted from the tissue/organ samples collected from individual mouse infected by HY12. Virus loads in different tissues of infected mice were quantitated with real-time PCR method using SYBR^®^ Green PCR Master Mix and SYBR^®^ Green RT-PCR Reagents Kit following the manufacturer’s instructions (Applied Biosystems, Foster City, CA, USA), and expressed as average virus copies of Log 10/g tissue.

## Results

### ICR murine strains is the most susceptible mice strain to HY12 infection

**Fig 1:**
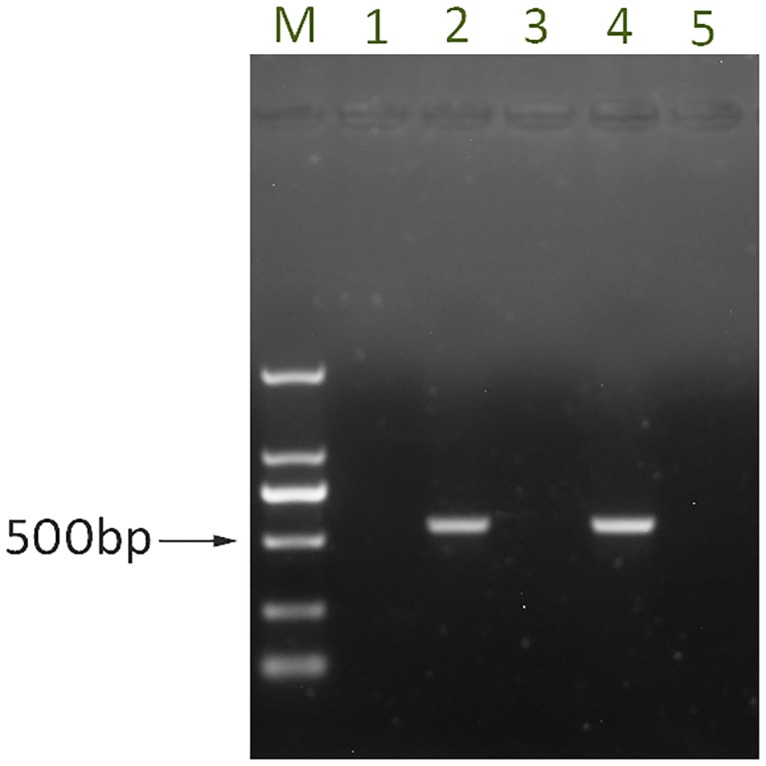
ICR suckling mice are susceptible to HY12 enterovirus infection. Three-day old Balb/c, Kunming, and IRC neonatal mice were subcutaneously inoculated with 2×10^6^ TCID_50_ HY12 viruses. Tissue samples including liver, lung, spleen, lymph node, kidney, and brain were collected from pups at 5 dpi and processed for amplification of HY12 genomic fragments using RT-PCR. Fragment with expected size was detected clearly from tissue samples in ICR (lane 2) suckling mice. No fragments were amplified from Balb/C suckling mice (lane 1) and Kunming suckling mice (lane 3) infected with HY12. Lane 4 and lane 5 were the positive and negative control, respectively. DNA ladder was presented as M and the size of expected fragment is indicated as arrow.

To determine the susceptibility of mouse strains to HY12 viruses, five neonatal mice at age of 3 days from the BALB/c, Kunming, and ICR strains were raised with the dam and infected subcutaneously with HY12 viruses. Each mouse was inoculated with 50 μl of viruses containing 2 × 10^8^ TCID_50_. Five days after infection, the mice were euthanatized and samples were collected and processed for RT-PCR amplification. As shown in Fig 1, fragments with expected size were detected in ICR mice, while no fragments were obtained in Balb/c mice and Kunming mice. Those results indicate that the neonatal ICR mice were susceptible to HY12 infection, and Balb/C and Kunming suckling mice were likely resistant to HY12 infections. Therefore, ICR mice were chosen in subsequent studies.

### Infective dose of HY12 viruses to ICR mice

**Fig 2:**
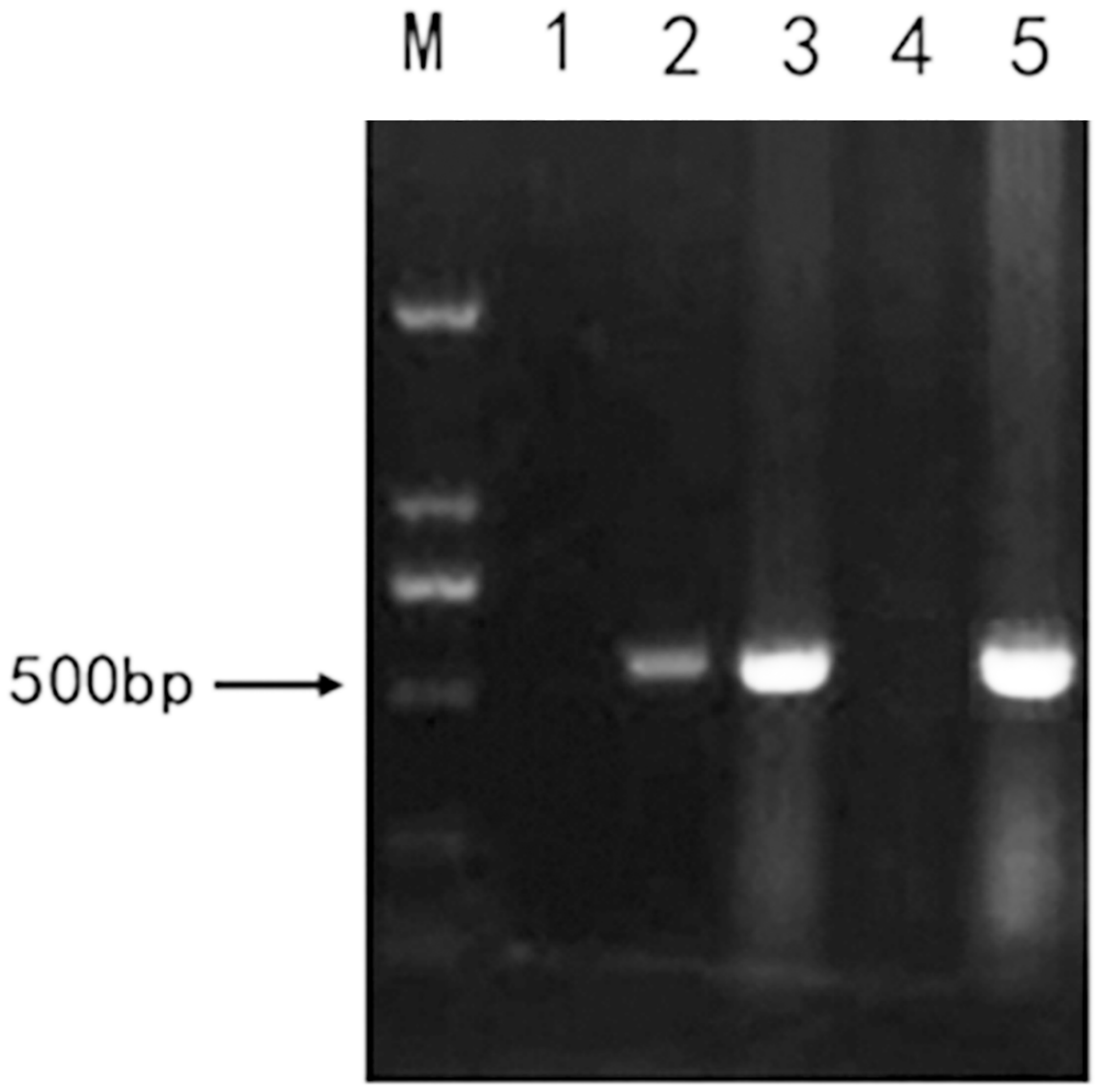
Minimal infective dose of HY12 to ICR suckling mice. 2 × 10^4^, 2 × 10^6^ and 2 × 10^8^ TCID_50_ HY12 viruses were injected to mice subcutaneously to determine the minimal infective dose (MID). Tissue samples were collected 5 dpi and processed for RT-PCR to detect the virus genome fragment. Representative figure showing the PCR-amplified fragments with expected size from mice infected with 2 × 10^6^ TCID_50_ (lane 2) and 2 × 10^8^ TCID_50_ (lane 3), respectively. The negative and positive controls were presented in lane 4 and lane 5, respectively. M stands for the DNA ladder.

To determine the minimal infective dose to ICR mice, different doses (2 × 10^4^, 2 × 10^6^ and 2 × 10^8^ TCID_50_) of HY12 viruses were subcutaneously administered to 5 mice per dose. Tissue samples including spleen, liver, lung, brain, and kidney from each mouse were collected after 5 dpi. 0.5 μg of the extracted RNAs from each organ above was used and processed for RT-PCR. As shown in Fig 2, fragments with expected size were amplified from 5 mice infected with 2 × 10^6^ TCID_50_ (lane 2) and 2 × 10^8^ TCID_50_ (lane 3), respectively, where no visible fragments were obtained in 5 mice infected with 2 × 10^4^ TCID_50_ (lane 1), demonstrating that the infective dose for HY12 viruses to infect ICR suckling mice is 2 × 10^6^ TCID_50_.

**Fig 3:**
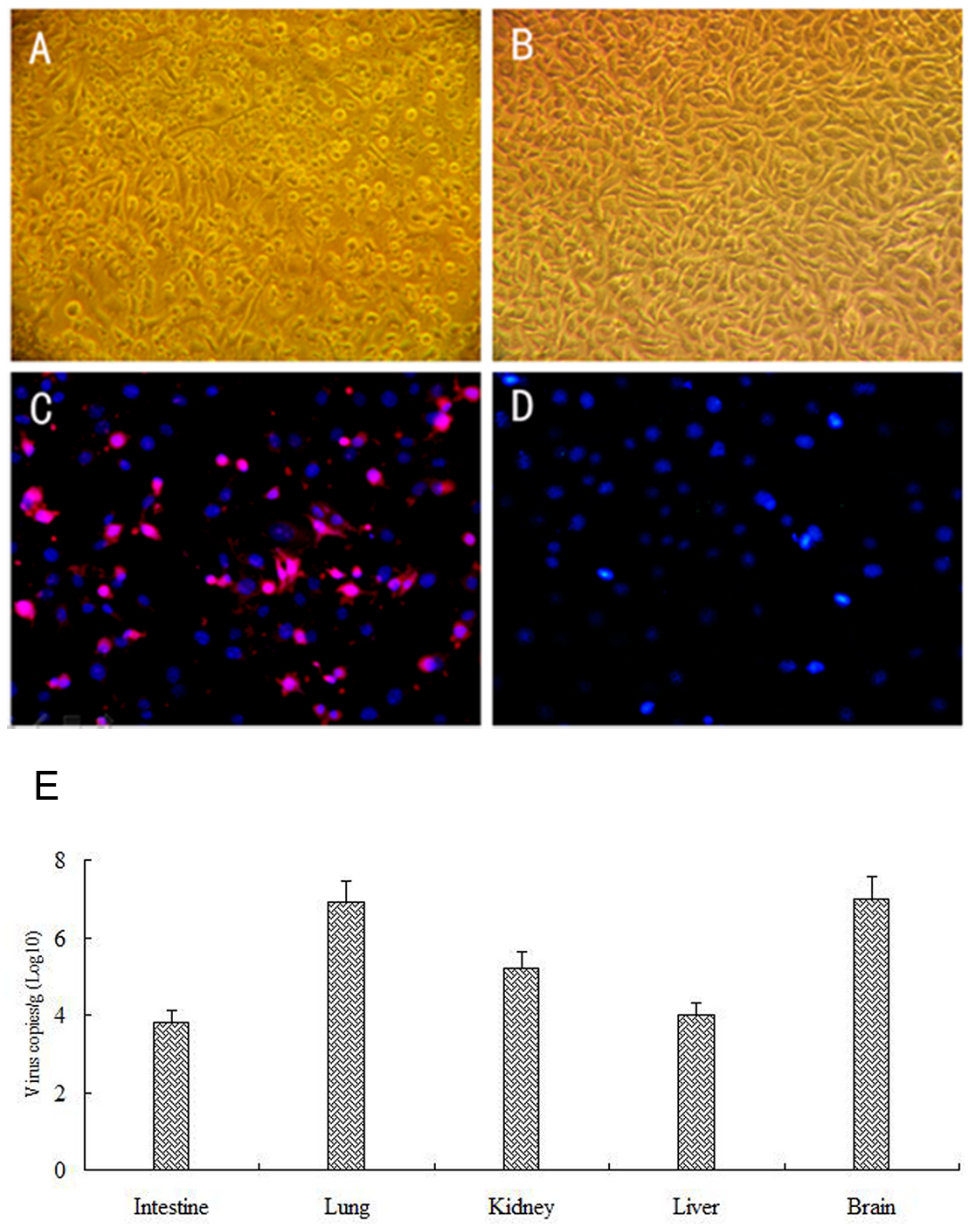
Confirmation of the HY12 infection by recovering the virus from the infected mice by HY12. Representative figure showing the cytopathic effects in Vero cells after they were infected by intestine tissue samples collected from mice infected with HY12 (A) in comparison with Vero cells infected with corresponding tissue samples from normal mice (B). Immunofluorescent assay was used to detect in HY12 infected cells using polyclonal antibody generated in rabbits against HY12 VP2 protein. Rhodamine-conjugated goat anti-rabbit IgG was used as secondary antibody. The Fluorescent signals were detected in HY12 infected cells (C) with the negative control cells (D). Viral loads in tissues for three mice infected by HY12 virus were quantitated and represented as average virus copies of log 10/ g tissue(E).

To assure the infection of HY12 viruses to suckling mice, same set of tissue samples collected at 5 dpi were also used for virus isolation. As shown in Fig 3, the Vero cells showed typical cytopathic effects after they were inoculated with HY12-infected mouse tissue samples (Fig 3A), while no CPEs were observed in Vero cells inoculated with normal mouse tissue samples (Fig 3B). Immunofluorescent assay revealed the strong fluorescent signals in the Vero cells infected with the recovered HY12 viruses (Fig 3C). No fluorescent signals were detected in control Vero cells (Fig 3D). Those results confirmed that ICR suckling mice were infected by HY12 viruses. Furthermore, quantitation of the virus loads in different organ samples using real-time PCR showed the virus loads in tissues of infected mice (Fig 3E).

Taken together, the above results demonstrated the infection of mice by HY12 viruses.

### HY12 viruses infect ICR suckling mice via multiple infection routes

**Fig 4:**
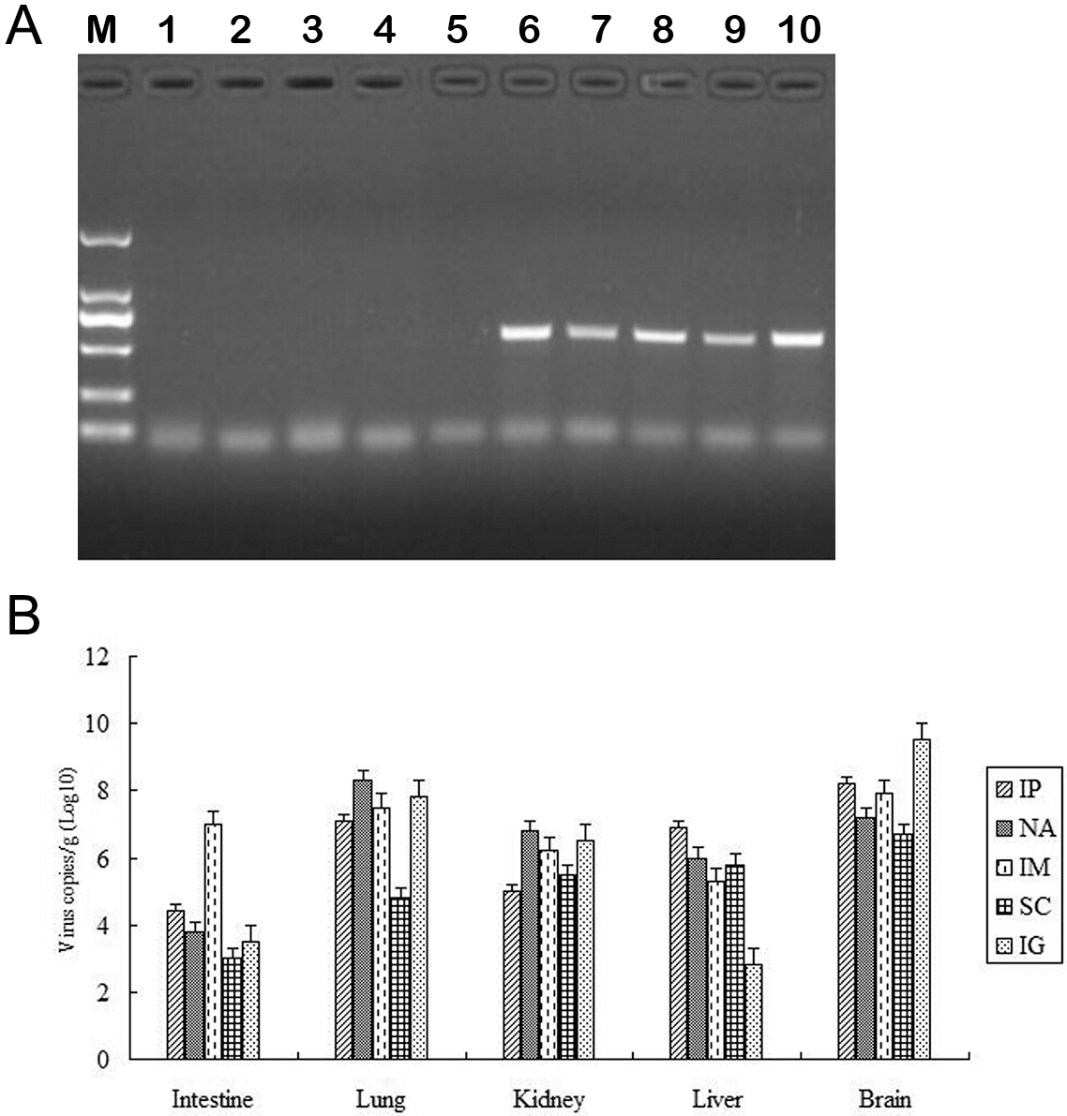
Determination of HY12 infective routes. ICR suckling mice were infected with HY12 virus by administered via intranasal, oral administration, intraperitoneal injection, subcutaneous injection and intramuscular injection, respectively. Tissue samples were collected and processed for RT-PCR to detect the virus genome fragments and virus loads in each organ was quantitated. Compared with the normal controls (lane 1–5), the fragments with expected size were amplified from tissues in mice infected with HY12 via all infection routes (lane 6–10). Lane 6: intranasal route; lane 7: oral administration; lane 8 intraperitoneal injection; lane 9: subcutaneous injection; lane 10: intramuscular injection. The virus loads in each organ were quantitated and represented as average virus copies of log 10/g tissues.

To determine the infection routes for HY12 viruses, 2 ×10^6^ TCID_50_ viruses (5^th^ passage) were administered to ICR suckling mice by intranasally, oral administration, intraperitoneal injection, subcutaneous injection and intramuscular injection, respectively, as described in material and methods. Five mice were administered for each route. Tissue samples from each mouse were collected after 5 dpi and processed for detection of virus. As illustrated in Fig 4A, no fragments were obtained in normal control mice (lane 1–5). However, fragments with expected size were amplified from tissues of mice infected with HY12 via all infection routes (lane 6–10), demonstrating that HY12 had the competence of infecting ICR suckling mice via multiple routes. To further confirm the above results, the virus loads in different organs for mice infected with HY12 via various infection routes were detected by real time PCR. As shown in Fig 4B, different titers of HY12 viruses were detected in almost every tissue of mice infected with HY12 viruses by different administration routes, further demonstrating that HY12 have the capability of infecting IRC mice via all routes.

### Growth inhibition in mice infected by HY12 viruses

**Fig 5:**
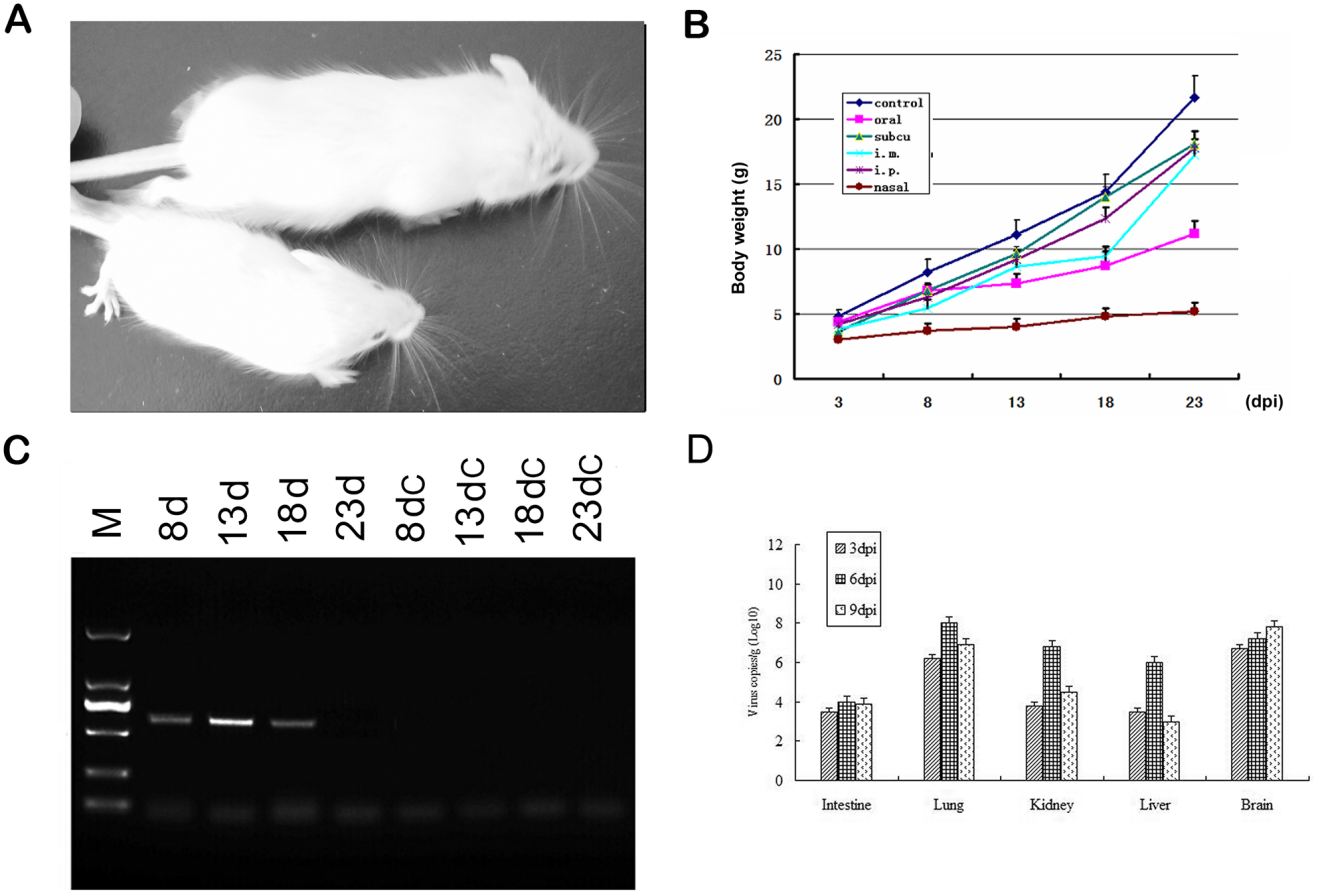
Effect of HY12 infection on mice. Mice were observed every 6 hours after they were infected by HY12 viruses. Body weights of the infected mice were weighed at interval of 5 days till endpoint (23 dpi). A representative figure showed the difference of mice infected with (lower) or without (upper) HY12 via intranasal administration (A). The average body weights in mice infected intranasally and orally were significantly lower than that in mice of the normal control (P<0.05) (B). HY12 viruses persistently present in the infected mice. Tissue samples in every mouse were collected at 3, 8, 13, and 18 dpi and viruses were still detected from infected mouse tissues 18 dpi using (C). Quantitation of virus loads in tissue from mice infected by HY12 at different days postinfection (D).

To determine the effect of HY12 infection, body weight for 5 mice were scaled at interval of 5 days till endpoint (23 dpi). As shown in Fig 5A, the mice infected with HY12 viruses showed a retard growth in comparison with the control group. The average body weights were significantly lower in mice infected intranasally and orally in relation to normal mice control (p<0.05) at time point of 8, 13, 18, 23 dpi. These results demonstrated that HY12 infections affect the normal growth of mice, especially when the viruses administered via intranasally or orally (Fig 5B).

To further ascertain the virus persistence and infection pattern in mice, tissue samples from each mouse were collected regularly from mice infected via intranasally and used for RT-PCR detection. As shown in Fig 5C, the viruses were detected continuously in mice infected with HY12 till 18 dpi, suggesting that HY12 persists in mice at least 18 days after they were infected.

Viral loads in tissues including lung, intestine, and brains of infected mice were quantitated as shown in Fig 5D. These results demonstrate infection of HY12 in mice model is persistent.

### Pathogenicity of HY12 virus to suckling mice

**Fig 6:**
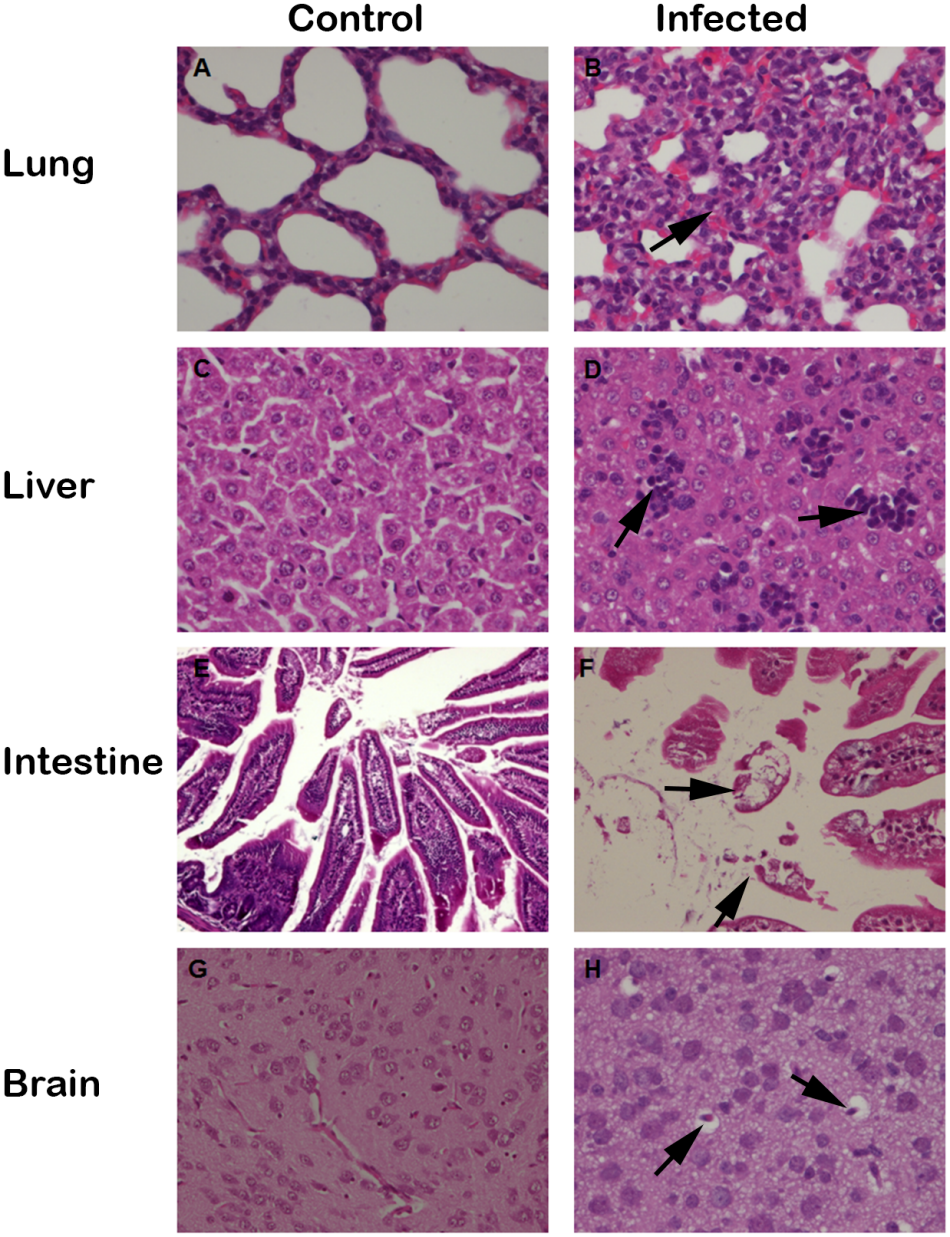
Histopathological lesion revealed in mice infected with HY12. Representative figures showing histopathological lesions in mice infected via oral administration. Tissue samples were collected and processed for H&E staining and histopathological examination. Obvious histopathological lesions and inflammatory cell infiltration were observed in the lung (B), liver (D), intestine (F) and brain (H) as indicated by arrow in comparison with corresponding tissues in uninfected control mice (A, C, E and G).

The above results demonstrated that HY12 infection had a significant effect on mice growth. To explore the pathogenicity of HY12 to ICR mice, tissues from mice infected by HY12 viruses via different infection routes and control groups were collected and processed for microscopic lesions examination. As shown in Fig 6, HY12 infection caused microscopic lesions extensively in tissues like lung, brain, liver and intestine. A huge amounts of inflammation cells infiltrated to the lung and resulted in the disruption and replacement of normal alveolar structure (Fig 6B). The inflammation infiltrates were also revealed in liver (Fig 6D). The epithelial villa in the small intestine were severely disrupted (Fig 6F). Cell degeneration and vaculation were observed in brain (Fig 6H). The pathological lesions observed in mice infected with HY12 through different infection routes were listed in Table 1.

**Table 1:** Histopathological lesions of mice experimentally infected with HY12 with different routes.

Tissue/inoculation	Intraperitoneally	intranasally	intramuscularly	subcutaneously	gavages
Small Intestine	**++**	**+++**	**+**	**+**	**++**
Lung	**+++**	**+++**	**+++**	**++**	**++**
Kidney	**+**	**++**	**+**	**++**	**++**
Liver	**+++**	**+**	**++**	**+++**	**+**
Brain	**+**	**+++**	**++**	**++**	**+**

“+” refers to light histopathological lesions; “++” stands for moderate histopathological lesions; “+++” refers to severe histopathological lesions. The results were the average of the five mice examined.

### Viral tropism in mice infected with HY12

**Fig 7:**
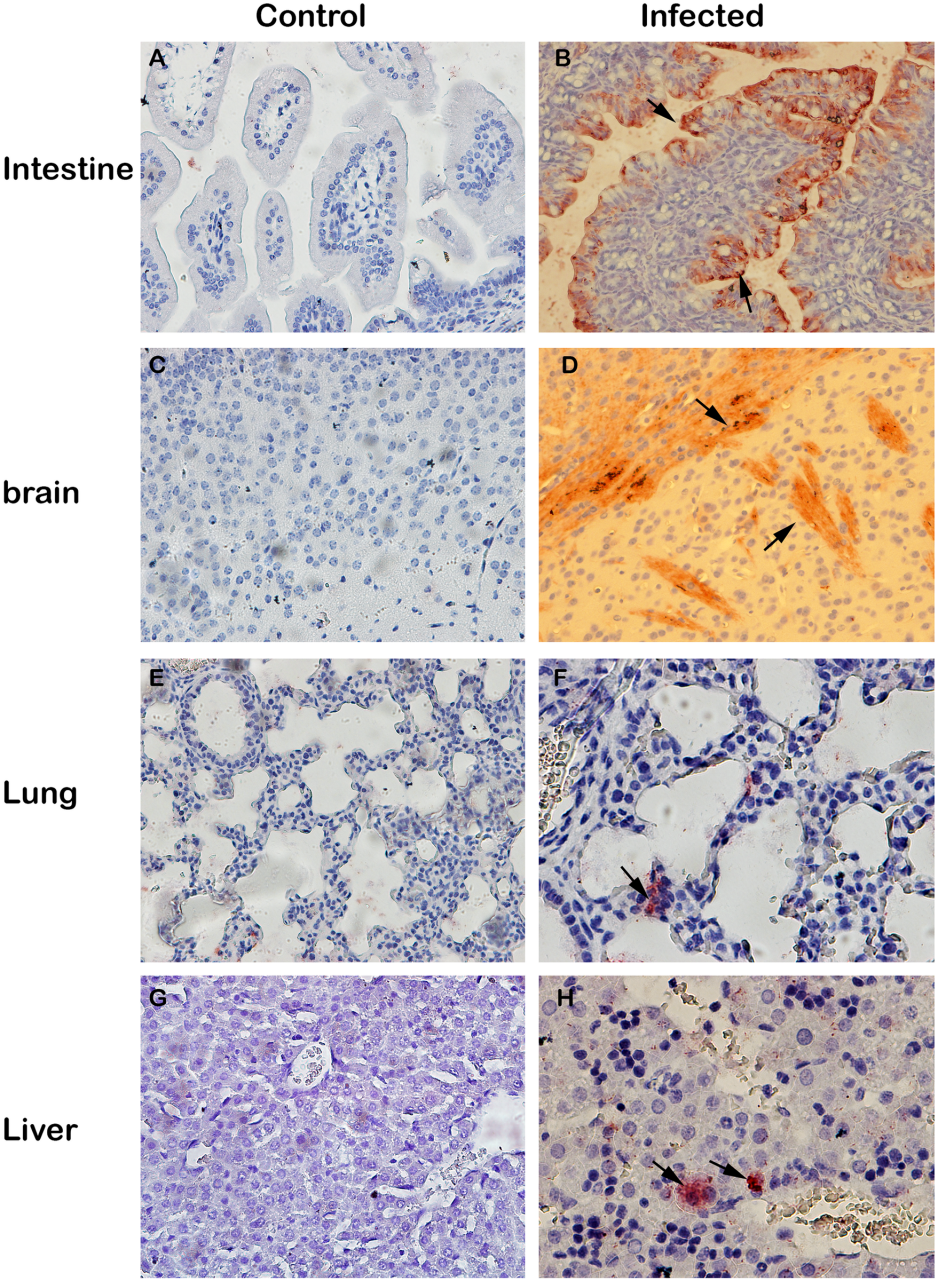
Viral tissue tropism in mice infected with HY12. Representative figure showing the antigen distribution in tissues of mice infected via oral administration. Tissues were collected and processed for immunohistochemistry assay to examine the tissue tropism of HY12 viruses. HY12 virus antigens were detected in the majority of the tissues examined, especially in the intestine(B), brain (D), lung (F) and Liver (H) as indicated by arrow in comparison to the corresponding tissue organs in noninfected mice (A, C, E and G).

To further define the viral antigen distribution and tissue tropisms for HY12, immunohistochemistry assay was employed to detect the virus antigen in mice infected by HY12 via various infection routes. Tissue samples including liver, lung, spleen, brain, kidney, intestine, and heart were examined by immunohistochemistry assay using the antibody generated against HY12 VP2 proteins. As illustrated in Fig 7, in comparison to the corresponding tissue organs in noninfected mice controls (Fig 7A and 7C), the virus antigens were revealed in the majority tissue of mice infected HY12, especially in tissues of intestine (Fig 7B), and brain (Fig 7D). The virus antigen distributions in mice infected by HY12 viruses via various routes were evaluated and summarized in Table 2.

**Table 2:** HY12 virus antigen detected in tissues in mice infected with HY12 by different routes.

Tissue/inoculation	Intraperitoneally	intranasally	intramuscularly	subcutaneously	gavages
Small Intestine	**++**	**++**	**+++**	**+**	**+**
Lung	**++**	**+++**	**++**	**+**	**++**
Kidney	**+**	**+++**	**++**	**++**	**++**
Liver	**+++**	**+**	**++**	**+++**	**+**
Brain	**+++**	**+++**	**++**	**++**	**++**

“+” refers to low rate of cells detected as HY12 positive; “++” stands for relative high rate of cells detected as HY12 positive; “+++” refers to many positive cells detected with HY12 antigen. The results were the average from five mice examined.

## Discussion

In a previous study, we reported the isolation and characterization of a novel enterovirus E isolate HY12 that is etiologically associated with an emerging infection in cattle characterized by severe respiratory and digestive disease with high morbidity and mortality rate in China [4]. To determine the pathogenicity of HY12 isolate, we employ HY12 viruses to experimentally infect different murine strains to establish a murine model system. The findings reveal the most susceptible mouse strain, the minimal infective dose, the infection routes, the pathological lesions, and tissue tropism, thus demonstrating that ICR mouse is the ideal model system for HY12 virus infection.

Murine model system has been widely used for the studies of human enterovirus infection [17–21]. However, this model system is barely reported for the studies of animal enterovirus infections. As enterovirus infects a variety of animal hosts and exhibits a diversified pathogenicity to animals, studying viral pathogenesis, especially enterovirus infection for large animals became very difficult due to shortage of eligible animals and concern of economic cost. The murine model system established in this study facilitated the studies for viral pathogenesis and immunity triggered by bovine enterovirus infection. The finding that HY12 viruses easily infected the neonatal ICR mice indicates ICR mouse is indeed an ideal model system to investigate the pathogenicity and the tissue tropism for HY12 viruses although there were curbs in administering virus to neonatal mice via intranasal and oral route. Revealing of the minimal infective dosage, the infection route, the pathological lesions and tissue tropism in the HY12 infected ICR mice ensured that the ICR mouse is an invaluable model system for future investigation of HY12-related pathogenesis and immune response.

Sample collection and processing has direct effect on the outcomes of the experiments. The samples used in this study were collected and processed based on issues addressed. For virus recovery and virus detection in HY12 infected mice, we utilized the procedure of initially mixing tissue samples from individual mouse to confirm virus infection. This simplified the procedure, saved time and effort without affecting the confirmation of virus existence. Although it lacks the ability to differentiate virus existence in concrete organs, the virus tropism determined by immunohistochemistry assay for the organs/tissues in each mouse guaranteed the detection of virus distribution and virus tropism, thus providing a reliable approach to detect viruses, especially for those of the neonatal suckling mice. For viral pathogenicity and pathogenesis studies, we collected individual organs/tissues to explore the viral tissue tropism, which ensured the unveiling of panorama of viral tropism. Based on our findings, we proposed that murine model system for HY12 infection should mainly include the following aspects: 1) the most susceptible murine strain is the neonatal ICR mouse; 2) although all infection routes are effective for HY12 infection, the intranasal administration is the best infection route for HY12 infection because it usually yield a severe clinical symptom and pathological lesions; 3) although HY12 had a pantropism to all tissues examined in the infected mice, the lung, intestine and brain are the organs/tissues with much severe lesions and viral antigen distribution, thus being as the target organs used to evaluate HY12 virus infections.

Enteroviruses have been demonstrated to be the etiological agents associated with the respiratory and digestive diseases in human and animals [3,4,11,13,16], and bovine enterovirus infection usually cause cattle showing clinical signs varying from respiratory, enteric and even infertility. Previously, we reported the identification of a novel enterovirus E isolate HY12 from the cattle showing severe respiratory and digestive disease with high morbidity and fatality rate. We found that HY12 possessed a unique biological characters and mutations its structure proteins. Recently, we identified a novel caprine enterovirus (CEV-JL14) from the severe diarrhea goats with a morbidity and mortality rate up to 84% and 54%, respectively. Those discoveries indicate that enteroviruses are likely etiologically associated with the severe digestive and respiratory diseases. The finding that HY12 enterovirus had the ability to infect neonatal ICR mice via multiple routes, the severe histopathological lesions in lung, intestine, and brain tissues suggest that HY12 viruses, like the other enterovirus, mainly targets the digestive, respiratory, and neurological system in neonatal model, which are congruent with the clinical observations on cattle naturally infected by HY12. The discovery that HY12 viral antigens were distributed in the majority of the examined tissues, especially in lung, intestine and brain tissues also support that neonatal ICR mouse is an ideal model system for exploring HY12-related viral pathogenesis and elicited immunity in the future.

Like the other enteroviruses, HY12 viruses harbor a single-stranded, positive RNA genome. The viruses hijack the transcription and translation system of host cells immediately upon infection and release of genomes into host cells to synthesize viral structural proteins and genomes, normally within 2–3 h. Detection of viruses by RT-PCR and virus isolation from the mixed samples collected in mice 5 dpi excludes the possibility that the viruses detected were the initially administered viruses, thus confirming the detected viruses were the progenies of the original administered viruses. The finding that viruses were detected in infected mice till 18 dpi confirmed the HY12 were indeed infected mice, which is also confirmed by the detection of virus antigen using immunohistochemistry assay.
